# Impaction of Rectum

**Published:** 1898-07

**Authors:** H. E. James

**Affiliations:** Government Veterinary Inspector, Ottawa, Canada


### IMPACTION OF RECTUM.

By H. E. James, V.S.,
GOVERNMENT VETERINARY INSPECTOR, OTTAWA, CANADA.

*Subject :* A four-year-old Clydesdale mare, weighing about 1600 pounds.

*History :* Seen first in May, 1897. Mare had been at pasture, and had been noticed very dull for several days, but no attention was paid to her until the day I was called, when colicky symptoms developed. Pasture was good and water abundant.

*Symptoms :* Pain very severe, the mare rolling violently and sweating freely. Examination revealed rectum impacted and enormously distended with feces. It required fifteen minutes' manual effort to remove sufficient to allow entrance of nozzle of syringe, after which an astounding amount of fecal matter was eliminated. A hypodermic injection of morphia was given, followed by an eight-drachm ball of aloes. Soft feed advised and regular exercise.

Two months later I was summoned again to see her, finding the same condition recurring, the impaction not so marked. At this time she was being worked and was fed on bran and oats, with hay and a bran-mash every Saturday night. The same treatment was applied, and, after relieving the acute symptoms, nux vomica was administered twice daily for several weeks and a pint of raw linseed-oil once a week.

Since the second attack I have been summoned four times to see her, finding her condition but slightly changed from the first attack. I have changed her feed, applied counter-irritation, given salines, with no better effect. She is now working in a lumber-team, and on an average the enema syringe is called into requisition every fortnight. Would be glad to hear of any similar case, the treatment pursued, and results obtained.

